# Independent third row augmentation of massive rotator cuff repairs: surgical technique with radiological and patient outcomes

**DOI:** 10.1016/j.xrrt.2024.12.011

**Published:** 2025-01-23

**Authors:** Samuel P. Mackenzie, Miloš Spasojevic, Travis Falconer, Lisa Kruse, Amy Randazzo, Codey Burton, Allan Young, Benjamin Cass

**Affiliations:** a Edinburgh Orthopaedics, Royal Infirmary of Edinburgh, Edinburgh, UK; b Sydney Shoulder Research Institute, St Leonards, NSW, Australia; c Perth Orthopaedics & Sports Medicine Center, West Perth, WA, Australia; d Department of Orthopedic Surgery, University of Wisconsin, Madison, WI, USA; e Department of Orthopaedic Surgery, Royal North Shore Hospital, St Leonards, NSW, Australia

**Keywords:** Rotator cuff repair, Rotator cuff augmentation, Triple row, Shoulder arthroscopy, Rotator cuff augment, Independent row

## Abstract

**Background:**

Primary repair of massive posterosuperior rotator cuff is challenging with a high rate of failure. This study details the technique and outcomes of a standard double-row cuff repair augmented with a synthetic ligament inserted in a separate adjacent location from the tendon-bone construct to off-load and de-tension the repair interface.

**Methods:**

Eleven patients with massive rotator cuff tears involving two or more tendons with >2.5 cm of retraction were prospectively enrolled. All patients underwent arthroscopically assisted mini-open double-row repair. This was augmented by a synthetic ligament passed medially through the supraspinatus and infraspinatus musculotendinous junctions before fixation distal to the second-row anchors. This represents a third row of repair that is remote from the double-row construct and aims to minimize repair tension. The primary outcome was repair integrity according to the Sugaya classification on postoperative magnetic resonance imaging. Secondary outcomes included the Constant score, EQ-5D 3L, and Oxford Shoulder Scores.

**Results:**

The mean patient age was 65 with 10 males. At a mean follow-up of 13 months, 8 (73%) of the repaired tendons were intact on magnetic resonance imaging. Of the 3 retears, one occurred at the musculotendinous junction. All outcome scores were significantly improved after surgery beyond the minimal clinically important difference.

**Conclusion:**

The insertion of an independent third row to off-load a standard rotator cuff repair construct resulted in favorable healing rates in patients with massive cuff tears. The technique is a simple, time-efficient method of de-tensioning the repair of massive rotator cuff tears.

Rotator cuff tears are a frequent cause of patient morbidity with a prevalence of approximately 20% in the general population.^49^ While the majority of tears can be managed nonoperatively, persistent pain and dysfunction can be addressed with surgery. Massive cuff tears, defined as those involving two or more tendons with >2.5 cm of medial retraction, draw particular attention due to the complexities of surgical fixation and variable patient outcome.^20^ Reducing tension at the tendon-bone interface is considered a key component to surgical success. Numerous techniques may help attain a tension-free repair and include capsular and subacromial releases, anterior and posterior slides, medialization of the cuff footprint, and extended postoperative immobilization in an abduction brace.^4^^,^^18^^,^^27^^,^^34^^,^^44^ Despite these efforts, retear rates as high as 94% have been reported, suggesting further innovation is required to improve outcome.^16^

Adjuncts to rotator cuff repair come in the form of synthetic ligaments, collagen matrices, and dermal allografts. These implants can function to reinforce a repair, offer a matrix for cell ingrowth, interpose a gap between the tendon and bone, or provide a tenodesis between the humerus and glenoid (superior capsule reconstruction).^3^^,^^5^^,^^15^^,^^16^^,^^35^^,^^38^ This study presents a new use for a synthetic ligament which focuses on reducing the tension of a double-row cuff repair with the ancillary benefits of potential cell ingrowth. A braided ligament is placed medial to the repair sutures and secured lateral to the second row anchors, thus providing support to the repair without being an integrated component of the tendon-bone construct. This is termed *independent third row augmentation*. The surgical technique is described, as are the radiological and functional outcomes of the initial cohort of patients treated with this method.

## Methods

### Patients

Eleven patients with massive rotator cuff tears confirmed on magnetic resonance imaging (MRI) as involving both the supraspinatus and infraspinatus with >2 cm of retraction were prospectively enrolled in the study.^11^^,^^17^ All patients had a history of trauma, and some reported preceding shoulder issues. Exclusions included Goutallier muscle grade >2, tears retracted beyond the glenoid margin, Hamada arthropathy grade >1, and previous surgery or joint infection. Patient demographics were obtained and all consented to participation in the study. An ethical approval was obtained prior to study commencement.

### Surgical technique

**Figure 1:**
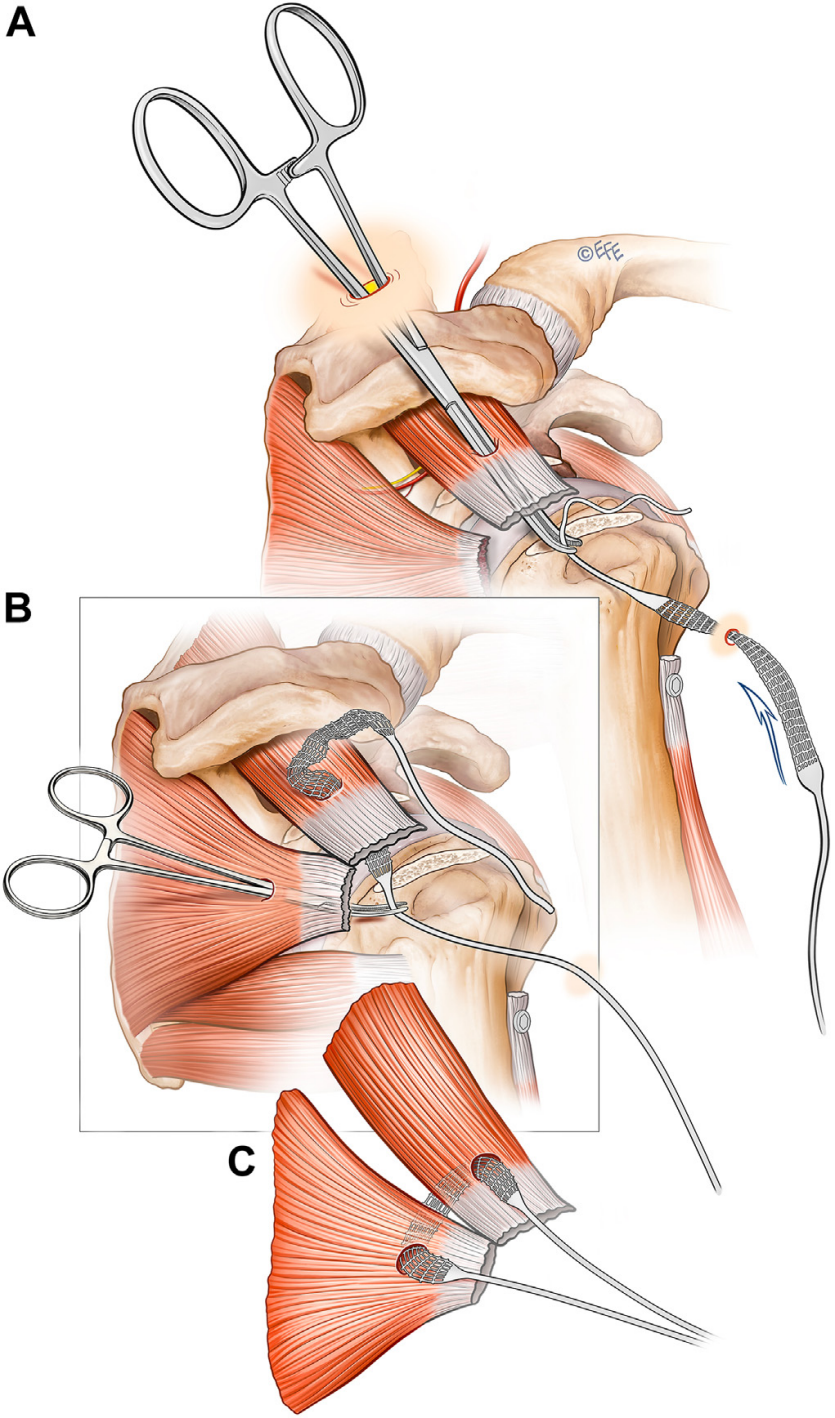
Ligament passage through the rotator cuff. (**A**) Retrograde through supraspinatus tendon from lateral portal, (**B**) grasping and passing of opposite ligament end through infraspinatus. (**C**) Tendon loop through musculotendinous junction of rotator cuff.

**Figure 2:**
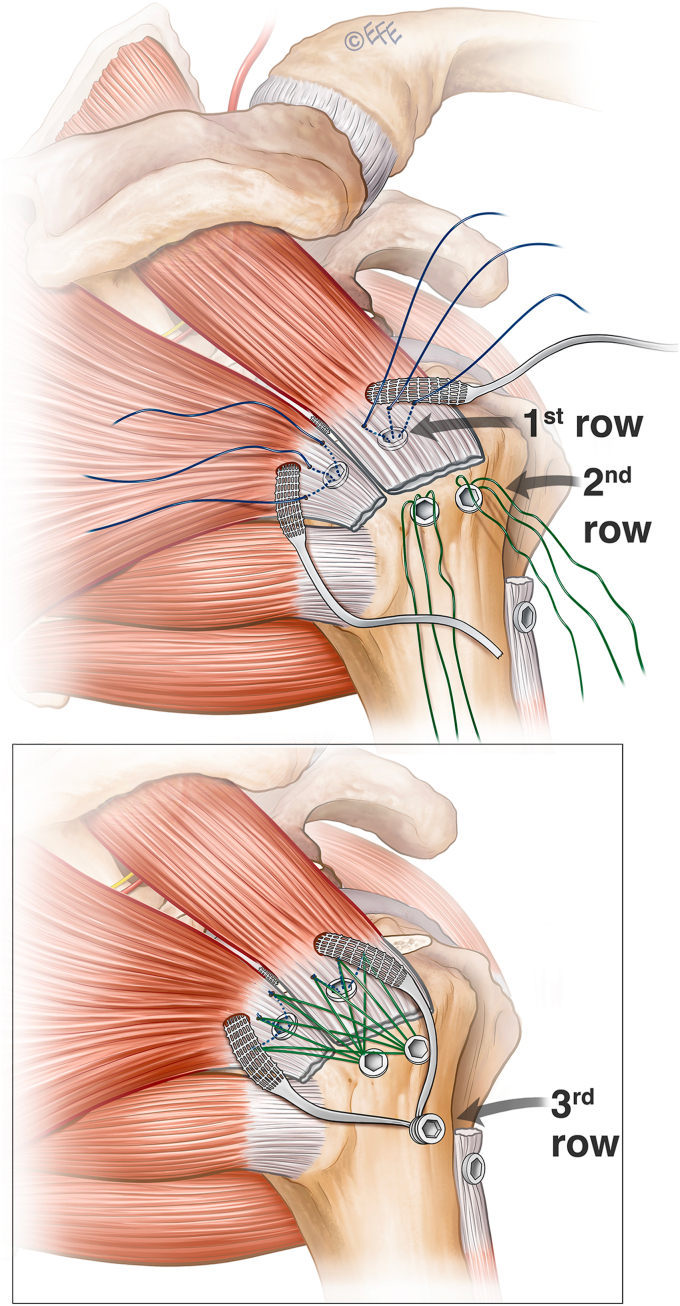
Anchor configuration; top image depicting situation prior to fixation of first and second row diamondback configuration. Lower image showing second step of third row fixation.

Independent third row augmentation is designed as a simple adjunct to a standard rotator cuff repair that can be planned preoperatively or added after initial arthroscopic inspection. It requires minimal additional soft tissue dissection or operative time. The technique detailed is performed in the lateral decubitus position but would be as simple in the beach chair setting. A standard posterior viewing portal is established to evaluate the glenohumeral joint. A lateral portal is established to allow instrumentation of the glenohumeral joint via the cuff tear for biceps tenotomy. The tear shape and size are assessed before performing an anterior interval slide. The coracoid is exposed in all cases to ensure a complete release of the coracohumeral ligament. The tear is assessed from the subacromial space, followed by bursectomy and subacromial decompression. Once the decision to augment the cuff repair with an independent third row is confirmed, the insertion of the synthetic ligament through the rotator cuff precedes completion of the definitive double-row repair. A modified Neviaser portal is created using an inside-out technique (Fig. 1). Through this portal a large arterial clamp pierces the musculotendinous junction of the supraspinatus. The synthetic ligament (Ligament Augmentation and Reconstruction System; Ligament Advanced Reinforcement System, Code R06x400/V, Ref L130605D; Corin Orthopaedics Holdings Limited, Cirencester, United Kingdom) is then passed retrograde from the lateral portal through the supraspinatus and out the Neviaser portal. An artery clamp then pierces the musculotendinous junction of the infraspinatus via the posterior portal and retrieves the second limb. Both limbs are then retrieved through the lateral wound to form an inverted mattress configuration which is secured laterally after the rotator cuff repair is complete. As per the usual preference of the senior author, the lateral portal is extended for a mini-open cuff repair. A standard double-row repair of the rotator cuff is performed using two triple-loaded medial anchors (Arthrex 3.5 mm double-loaded Corkscrew; Arthrex, Naples, FL, USA) with mattress sutures passed, tied, and incorporated in an alternating fashion to two lateral row anchors (4.75 mm Swivelock; Arthrex, Naples, FL, USA) to create a modified diamondback configuration (Fig. 2). A separate anchor is used to tenodese the biceps in the groove. Finally, the third row is completed by anchoring the two ends of the synthetic ligament lateral (distal) to the second row with a biceps tenodesis screw (7 mm Biceps tenodiesis anchor; Arthrex, Naples, FL, USA).

All patients are placed in a sling for six weeks, which is removed three times a day for hygiene purposes and to perform elbow range of movement exercises. At six weeks, patients are permitted to perform active movements as pain allows, with some gentle active-assisted flexion and external rotation. Active strengthening exercises are commenced at three months.

### Radiological and patient-reported outcome

All patients were evaluated with preoperative MRI which was repeated at a minimum of six months after surgery. Preoperative scans were measured for coronal retraction and sagittal extent. The muscles of the supraspinatus and infraspinatus were graded according to Goutallier.^31^ The presence of any subscapularis tear was graded according to Lafosse.^24^ Postoperative scans were graded according to Sugaya with grades 1-3 deemed “intact; and grades 4-5 “failed.”^42^ The failures were further graded according to Cho with a type one failure defined as those that occurred at the bone-tendon interface whereas type two failures occurred at the musculotendinous junction.^7^ Patient reported outcome measures including the Constant score, Oxford Shoulder Score (OSS) and the Euro-Qol 5D, and the EQ-5D visual analog scale health were performed 2 weeks before surgery and one week after postoperative MRI.^10^^,^^12^^,^^37^

## Results

**Figure 3:**
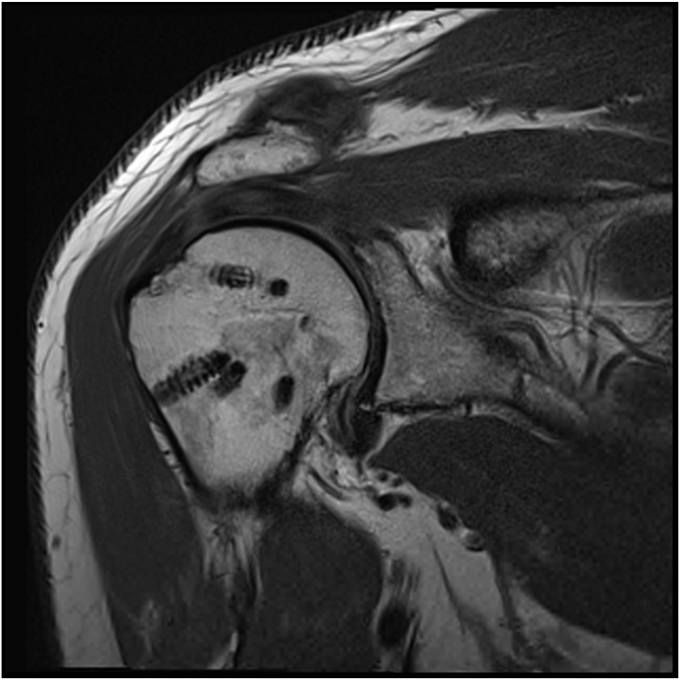
Healed massive cuff repair (Sugaya grade I) of patient 11. MRI performed 6 months after surgery.

**Figure 4:**
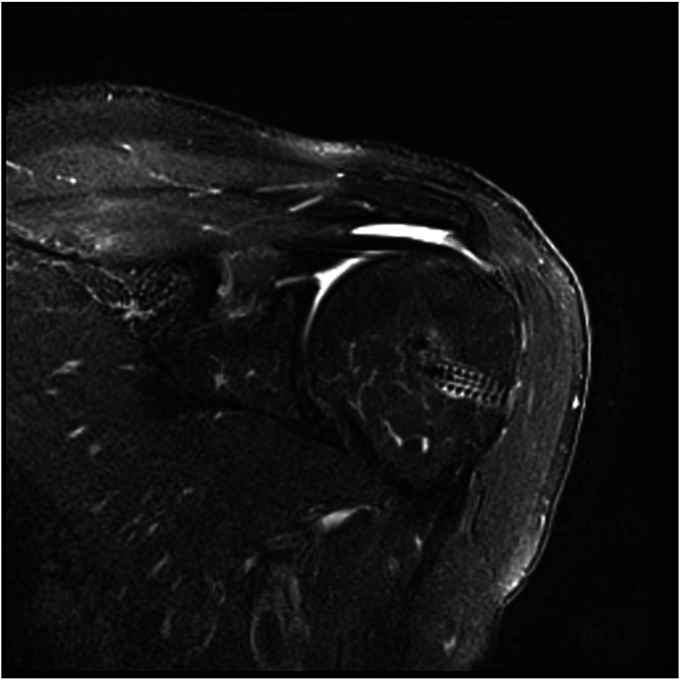
Healed massive cuff repair with thinning of the tendon (Sugaya grade III) of patient 8. MRI performed 10 months after surgery.

**Figure 5:**
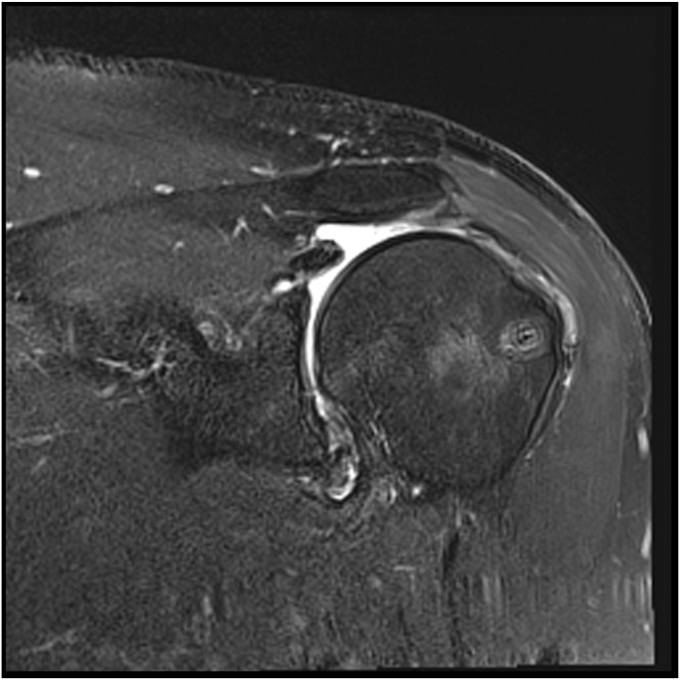
Failed rotator cuff repair at the musculotendinous junction of patient 18. MRI performed 8 months after surgery.

The study group was comprised of 10 males and one female with a mean age of 65 (range, 55-73). The preoperative characteristics for each cuff tear are detailed in Table I. The mean retraction was 28 millimeters. The mean time to postoperative MRI and follow-up was 10.7 months (range, 6-22). At the time of postoperative MRI, eight repairs were intact and three failed. Sugaya grading is included in Table I. Of the three failed repairs, one was graded as a type II (musculotendinous junction failure). Figure 3 shows an intact repair 13 months postsurgery (Sugaya grade I) and Figure 4 shows an intact but thinned rotator cuff repair at 23 months (Sugaya grade II). Figure 5 shows a failed repair with rupture at the musculotendinous junction at eight months (Cho type II and Sugaya V). All patient-reported outcomes improved significantly after surgery (Table II). Failure of the repair did not result in significantly lower mean change in Constant score (*P* = .2) or OSS (*P* = .15)

**Table I:** Preoperative and postoperative MRI grades of rotator cuff tears treated with double-row repair augmented with an independent third row.

Patient	Age	Preoperative MRI					Postoperative MRI	
		Coronal retraction (mm)	Sagittal extent (mm)	Goutallier grade of supraspinatus	Goutallier grade of infraspinatus	Lafosse grade of subscapularis	Intact (Y/N)	Sugaya classification
1	56	26	25	1	1	3	No	4
2	59	31	27	1	1	0	Yes	3
3	65	25	16	1	0	I	Yes	2
4	62	32	21	0	1	0	No	4
5	66	27	30	1	1	0	Yes	2
6	55	30	28	1	1	2	Yes	2
7	60	25	40	2	1	0	Yes	2
8	76	23	21	1	0	0	Yes	3
9	67	40	30	1	1	0	No	4
10	73	25	22	0	1	1	Yes	1
11	73	30	30	1	1	1	Yes	1

**Table II:** Patient-reported outcome measures before and after double-row cuff repair augmented with an independent third row.

	Preoperative (mean ± SD)	Postoperative (mean ± SD)	*P* value
Constant score	26.8 ± 7.0	77.1 ± 5.0	<.001∗
OSS	28.7 ± 7.4	44.2 ± 2.6	.002∗
EQ-5D	0.65 ± 0.05	0.91 ± 0.1	<.001∗
Health VAS	63.7 ± 7.4	85.5 ± 11.3	<.001∗

*VAS*, visual analog scale.

∗ Statistically significant *P* < .05.

## Discussion

The secure reconstitution of the tendon-bone interface is a key component in the surgical management of massive rotator cuff repairs. Despite various techniques to ensure appropriate tendon excursion, undue tension at the repair interface remains a cause of failure. Tension may be increased through the well-intentioned desire to compress the tendon over a large area of the tuberosity in an effort to improve healing. The independent third row acts much like a tension band cerclage wire/suture after patella tendon repair, a restraint added out with the primary surgical focus to minimize forces across the repair site. The reasoning behind the sequence of repair was to allow for a reproducible technique with good visualization and tensioning of the double-row first and off-load tensioning of the independent row second.

The described technique does not require planning prior to the case and can be added at any time before the repair is secured. The third row is simple, adds little surgical time, and is composed of implants commonly found in most orthopedic departments. Furthermore, we feel that a degree of variation from the implants prescribed in this article, whether it be due to surgeon preference or cost, would likely provide similar results.

The small prospective case series included in this study demonstrated improvement in both the Constant score and OSS well above the minimal clinically important difference. This is in keeping with many other studies which show favorable outcome after rotator cuff repair, and more speaks to the expected outcome of a healed tear than it does to the impact of a particular technique. Less commonly reported in rotator cuff studies was the improvement in health scores. Both the Euro-Qol 5D and visual analog scale health significantly improved after surgery, demonstrating the value of this intervention to a patient’s general health. The retear rate of 27% is comparable to other reports of similar pathology (general retear rates of 50%-53%, allograft/autograft 23.6%).^16^^,^^21^^,^^32^^,^^47^ The wide variation in reported success is most likely due to two factors: transparency in the parameters used to define large to massive tears and the timing and modality of postoperative healing assessment. For the purposes of this study, we defined such tears as those involving two tendons with retraction of at least 2.5 cm. Based on previous randomized trials, we hope that by reporting the dimensions of each tear, we add clarity and improve the transferability of our results. We chose postoperative MRI to assess healing to allow any failures to be identified and characterized.

Augmentations to optimize the mechanical durability of rotator cuff repair constructs can include devices that aim to load share, increase load to failure, decrease gap formation, and increase cuff footprint compression. This can be achieved with additional points of fixation and may be accompanied by grafts of varying constitution. Local autograft patches most commonly employ the long head of the biceps tendon whether it be incorporated into a repair or detached and used as a separate graft. Although reviews report 82% healing rates, the literature is limited to case series, heterogeneous rotator cuff tear groups, and varying techniques.^45^ Healing rates as low as 24%-45% are reported despite possible biologic advantages of this technique.^8^^,^^39^^,^^41^ Patches from other host sites such as the fascia latae can add mechanical strength to rotator cuff repairs in biomechanical studies but are more commonly used in nonanatomical procedures such as the superior capsular reconstruction.^22^^,^^30^ Graft retear rates range from 5% to 32%, while donor site morbidity and increased surgical time remain an issue.^6^^,^^25^^,^^26^^,^^33^^,^^40^ Allografts come in varying forms and their use as an interposition graft is supported by level I evidence in the management of massive rotator cuff tears with superior retear rates were significantly (15% vs. 60%) accompanied by better clinical outcomes.^1^^,^^23^ Xenografts may also be used as a bio-inductive graft to compliment a tenuous repair.^2^^,^^43^

Synthetic grafts, such as the Ligament Advanced Reinforcement System included in the present study, are developed to compliment a surgical repair. Due to the high variance in scaffolding type, there are a variety of synthetic products on the market, with variable retear rates between 10% and 62%.^9^^,^^13^^,^^28^^,^^36^ Knowledge of the optimal construct for rotator cuff repair is unclear. Biomechanical animal models suggest promising utilization for ligament reconstruction and augmentations, with similar tension failure loads to autologous tendon grafts, but lower quality than original natural tendon anatomy.^46^^,^^48^ Technical advances in soft tissue engineering currently allow molding of scaffolds at a nano level and in vitro evidence suggests the scaffold configuration influences biological healing responses.^14^ Further in vivo studies will be needed to guide surgical use in the future.

The main limitation of this study is the small number of included patients, and as such results may suffer from small sample size bias, thus small changes in failure rates would significantly alter the overall healing rate and outcome of this study. Although only 11 patients are included, we hope the prospectively collected radiographic and patient-reported data add credence to the description of our technique and its potential application.

Timing of follow-up MRI was variable, but all scans occurred at least six months postoperatively and would likely identify most failures.^19^^,^^29^ The technique described is simple, but the additional implant cost should be considered and may limit use in certain resource-constrained environments. Lastly, there may be some concern regarding the passage of a synthetic ligament through the musculotendinous junction and the potential for failure in this region; however, this is not exclusive to our technique and there were insufficient number of failures to draw conclusion.

## Conclusion

Independent third row augmentation of rotator cuff repairs offers a simple, time-efficient means of supporting and off-loading the surgical construct. The technique has shown favorable healing rates and patient-reported outcomes in a small prospective case series of patients with massive tears. Further research is required to elucidate the outcome of this technique in a larger cohort of patients with massive tears and to assess its potential in other settings such as revision surgery.

## Disclaimers:

Funding: No funding was disclosed by the authors.

Conflicts of interest: The authors, their immediate families, and any research foundation with which they are affiliated have not received any financial payments or other benefits from any commercial entity related to the subject of this article.
